# Pharmacological reversal of endothelin-1 mediated constriction of the spiral modiolar artery: a potential new treatment for sudden sensorineural hearing loss

**DOI:** 10.1186/1472-6815-5-10

**Published:** 2005-11-29

**Authors:** Elias Q Scherer, Wolfgang Arnold, Philine Wangemann

**Affiliations:** 1 Cell Physiology Laboratory, Dept. Anatomy & Physiology, Kansas State University, Manhattan, KS 66506, USA; 2 Department of Otorhinolaryngology, Head and Neck Surgery, Technical University of Munich, Klinikum rechts der Isar, Ismaninger Str. 22, 81675 Munich, Germany

## Abstract

**Background:**

Vasospasm of the spiral modiolar artery (SMA) may cause ischemic stroke of the inner ear. Endothelin-1 (ET-1) induces a strong, long-lasting constriction of the SMA by increasing contractile apparatus Ca^2+ ^sensitivity via Rho-kinase. We therefore tested several Rho-kinase inhibitors and a cell-permeable analogue of cAMP (dbcAMP) for their ability to reverse ET-1-induced constriction and Ca^2+^-sensitization.

**Methods:**

The present study employed SMA isolated from gerbil temporal bones. Ca^2+^sensitivity was evaluated by correlating vascular diameter and smooth muscle cell [Ca^2+^]_i_, measured by fluo-4-microfluorometry and videomicroscopy.

**Results:**

The Rho-kinase inhibitors Y-27632, fasudil, and hydroxy-fasudil reversed ET-1-induced vasoconstriction with an *IC_50 _*of 3, 15, and 111 μmol/L, respectively. DbcAMP stimulated a dose-dependent vasodilation (*Ec_50 _*= 1 mmol/L) and a reduction of [Ca^2+^]_i _(*EC_50 _*= 0.3 μmol/L) of ET-1-preconstricted vessels (1 nmol/L). Fasudil and dbcAMP both reversed the ET-1-induced increase in Ca^2+ ^sensitivity.

**Conclusion:**

Rho-kinase inhibition and dbcAMP reversed ET-1-induced vasoconstriction and Ca^2+^-sensitization. Therefore, Rho-kinase inhibitors or cAMP modulators could possess promise as pharmacological tools for the treatment of ET-1-induced constriction, ischemic stroke and sudden hearing loss.

## Background

The inner ear's blood supply depends solely on the spiral modiolar artery (SMA), a functional end artery. Vasospasm/constriction of the SMA can cause an ischemic stroke of the inner ear, leading to sudden sensorineural hearing loss (SSHL). Thus, investigating the mechanisms controlling the inner ear microcirculation is a prerequisite for the development of new strategies to treat SSHL.

Capillary blood flow is primarily regulated by the resistance of precapillary arteries. The vascular resistance is a function of the contractile status of the vascular smooth muscle cells (VSMCs). Constriction of VSMCs results from an increase in intracellular Ca^2+ ^([Ca^2+^]_i_) and/or by an increase in the Ca^2+ ^sensitivity of the contractile apparatus [1,2]. One key mechanism enhancing Ca^2+ ^sensitivity and thus vascular tone is Rho-kinase signalling, which results in inhibition of myosin light chain phosphatase [2,3]. Rho-kinase activation has been shown to cause vasospasm of coronary, cerebral and spiral modiolar arteries [4-9].

One of the strongest Rho-kinase activators described so far is the vasoconstrictor endothelin-1 (ET-1). The synthesis of ET-1 by endothelial cells is activated by physiological stimuli such as shear stress, insulin, thrombin and other vascular factors [10]. ET-1 and ET_A _receptors play a fundamental role in the maintenance of basal vasomotor tone in resistance arteries [11]. The synthesis of ET-1 can be increased by hypoxia and elevated oxidized low-density lipoproteins [12,13] and has been implicated in the pathogenesis of a number of cerebrovascular disorders, including stroke, ischemia, and, in particular, cerebral vasospasm [14,15]. Thus, ET-1 possesses pathological potential in addition to its physiological functions. ET-1 is present in the SMA and induces strong, long-lasting constriction via ET_A_-receptor-mediated Rho-kinase activation [9,16,17]. Taken together ET-1 is likely an endogenous regulator of inner ear microvascular tone.

We have previously shown that CGRP is able to reverse ET-1-induced constrictions in the SMA via an increase in vascular smooth muscle cAMP [18]. CGRP is present in perivascular nerves of the SMA and therefore is a potential endogenous vasodilator of the SMA. We propose, therefore, that reversal of ET-1-induced constriction is not necessarily limited to inhibition of ET-1-related mechanisms (e.g., Rho-kinase signalling).

These findings provide a clinical perspective for a new treatment of SSHL, because both Rho-kinase signalling and cAMP can be targeted via pharmacological agents. Therefore, we assessed the potency of clinically relevant Rho-kinase inhibitors and a cell-permeable analogue cAMP (dbcAMP) in terms of reversing ET-1-induced constriction and Ca^2+^-sensitization in the SMA.

## Methods

### Drugs and solutions

The physiologic salt solution (PSS) contained (in mmol/L) 150 NaCl, 3.6 KC1, 1.0 MgCl_2_, 1.0 CaCl_2_, 5.0 HEPES, and 5.0 glucose, pH 7.4. Extracellular Ca^2+ ^concentration ([Ca^2+^]_ex_) was raised to 3 and 10 mmol/L by addition of CaCl_2_. A maximal vasodilation was induced by the removal of extracellular Ca^2+^. The nominally Ca^2+^-free solution contained (in mmol/L) 150 NaCl, 3.6 KC1, 1.0 MgCl_2_, 1.0 EGTA, 5.0 HEPES, and 5.0 glucose, pH = 7.4. Fluo-4-AM (Molecular Probes) was dissolved in anhydrous DMSO and stored in 1 mmol/L aliquots. Y-27632 was kindly provided by Welfide. Fasudil was obtained from Calbiochem. Fasudil (obtained from Tocris Cookson) was modified to hydroxyfasudil by Dr. Duy Hua, Dept of Chemistry, Kansas State University. All other chemicals were obtained from Sigma.

### Preparation of the spiral modiolar artery (SMA)

Experiments were conducted on tissues isolated from gerbils under a protocol that was approved by the Institutional Animal Care and Use Committee at Kansas State University. Gerbils were anesthetized with sodium pentobarbital (100 mg/kg i.p.) and decapitated. Temporal bones were removed, opened and placed into a micro-dissection chamber containing PSS at 4°C. The SMA was isolated from the cochlea by micro-dissection as described previously [19]. Briefly, the cochlea was opened. The bone surrounding the modiolus was carefully removed and the SMA, which is only loosely attached to the eighth cranial nerve, was isolated. Care was taken to not stretch the artery.

### Simultaneous measurement of vascular diameter and [Ca^2+^]_i_

The simultaneous measurement of vascular diameter and [Ca^2+^]_i _has been described previously [17]. Briefly, the smooth muscle cells of vessel segments were loaded with the Ca^2+ ^indicator dye fluo-4 by incubation in PSS containing 5 μmol/L fluo-4-AM for 35 min at 37°C. After loading, vessel segments were washed with PSS and maintained at 4°C for 20 minutes prior to experimentation at 37°C. Vessel segments were transferred into a bath chamber mounted on the stage of an inverted microscope (Nikon). Fluorescence emitted by fluo-4 (518–542 nm) in response to excitation at 488 nm (Photon Technology International) was detected by a photon counter (Photon Technology International). For measurements of the vascular diameter, the vessel was illuminated at 605–615 nm and the transmission image was recorded with a chilled CCD camera (Hamamatsu). The outer vascular diameter was measured by two video edge detectors (Crescent). Fluorescence and calibrated diameter signals were digitized and recorded simultaneously (Photon Technology International).

### Experimental protocols

Experiments were started 20 min after loading with fluo-4. Vessel segments were superfused at a rate of 9 ml/min with PSS. This flow rate corresponds to an exchange rate of 2 bath chambers volumes/sec, given the bath chamber volume of 75 μl. Upon start of the superfusion, the unpressurized artery develops a spontaneous vascular tone that is sensitive to removal of extracellular Ca^2+ ^and inhibition of L-type Ca^2+ ^channels with nanomolar concentrations of nifedipine [19]. The viability of each vessel was assessed by its constrictor response to 10 mmol/L [Ca^2+^]_ex_. The [Ca^2+^]_i _was monitored as fluorescence intensity and was normalized to the basal fluorescent emission prior to the beginning of each experiment. The fluorescence and diameter values taken for statistical analysis represent averages of the [Ca^2+^]_i_-fluorescence and the vascular diameter over 30 sec beginning 30 sec after the onset of stimulation. We carefully validated the [Ca^2+^]_i _-measurements with the non-ratiometric Ca^2+^-dye fluo-4 by (i) excluding artefacts due to vessel diameter changes (evaluated by [Ca^2+^]_ex_-versus ET-1 dose-response curves), (ii) excluding significant differences in the magnitude of [Ca^2+^]_i _-changes between preparations, and (iii) assessing the same optimal dye-loading conditions in each experiment [9,17].

Affinity constants (K_DB_) and concentrations that cause a half-maximal inhibition (IC_50_) were determined in cumulative experiments and averaged after logarithmic transformation (p*K*_*DB *_and p*IC_50_*) as previously described [17].

The Ca^2+ ^sensitivity of the contractile apparatus was determined by a correlation of [Ca^2+^]_i _and the vascular diameter as described previously [9]. Changes in [Ca^2+^]_i _were induced by changes in [Ca^2+^]_ex_. Stepwise increases in [Ca^2+^]_ex _from 0 to 1, 3 and 10 mmol/L caused increases in [Ca^2+^]_i _and decreases in the vascular diameter. Correlations were found to be linear (*r *> 0.95) within the measured range (the relation between calcium concentration and tension gets sigmoidal if the [Ca^2+^]_ex _and thus the [Ca^2+^]_i _is further increased [9]). Slopes were quantified in the arbitrary unit μm/Ca^2+^, where μm represent the change in the vascular diameter and Ca^2+ ^represents the normalized change in the cytosolic Ca^2+ ^concentration. Linear slopes were obtained to compare the Ca^2+ ^sensitivity within paired experiments. In each vessel segment, the Ca^2+ ^sensitivity was assessed under control and under experimental conditions.

Experiments of dbcAMP induced dilations were bracketed by Ca^2+ ^free manoeuvres that were performed to induce a maximal vasodilation and reduce the [Ca^2+^]_i _to a minimal level. Vascular diameter or fluorescence intensity in the absence of Ca^2+ ^was considered as baseline. The magnitude of the vascular tone or fluorescence intensity was determined as the difference between the recorded value and the baseline value. Measurements were averaged over a period of 1 min to average vasomotion. DbcAMP-induced effects were normalized to the magnitude of the vascular tone or fluorescence intensity during 1 min immediately prior to the application of dbcAMP.

### Statistical analysis

All results are expressed as average ± SEM of n experiments with n representing the number of vessel segments. The significance of changes in the vascular diameter and of changes in the Ca^2+ ^sensitivity were determined using Student's paired *t*-test. Differences were considered to be significant at error probabilities less than 0.05 (*P *< 0.05).

## Results

This report is based on recordings of 50 vessels from 34 animals. The average vascular diameter was 65 ± 1 μm.

### ET-1-induced constriction is reversed via Rho-kinase inhibitors

**Figure 1:**
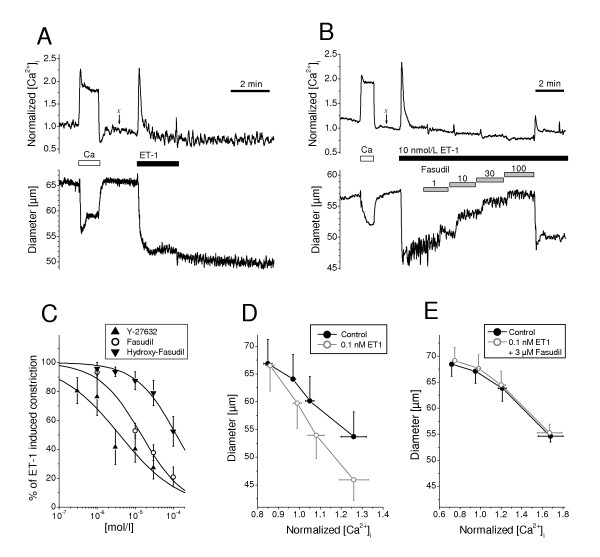
**Rho-kinase inhibitors reverse ET-1-induced constriction. A) **Effect of 10 nmol/L endothelin-1 (ET-1) on the cytosolic Ca^2+ ^concentration ([Ca^2+^]_i_) and the vascular diameter of the spiral modiolar artery. Note that ET-1 caused a transient increase in [Ca^2+^]_i _and a sustained vasoconstriction and increase in vasomotion. This constriction outlasted the stimulation with ET-1. Increasing extracellular Ca^2+ ^concentration ([Ca^2+^]_ex_) to 10 mmol/L ("Ca") induced an increase in [Ca^2+^]_i _with parallel vasoconstriction. This increase of the [Ca^2+^]_ex _from 1 to 10 mmol/L ("Ca") served as a control experiment. Measurements of [Ca^2+^]_i _were normalized to the value obtained prior to the admission of ET-1 (value at time '*x*' was set to 1). **B) **In the presence of 10 nmol/L ET-1, increasing concentrations of fasudil (1–100 μmol/L) induce dose-dependent relaxation. [Ca^2+^]_i _values were normalized, with the baseline value prior to application of ET-1 designated as 1.0 (denoted as "*x*"). **C**) Dose-response curves for Y-27632-, fasudil- and hydroxy-fasudil-induced reversal of ET-1 (10 nmol/L) -mediated vasoconstriction. **C) **ET-1 stimulated a significant increase in VSMC contractile apparatus Ca^2+ ^sensitivity. **D) **Fasudil completely reversed the ET-1-induced Ca^2+^-sensitization.

ET-1 (10 nmol/L) induced a transient increase in [Ca^2+^]_i_, a strong and long-lasting vasoconstriction and a robust increase in the vasomotion of the gerbil spiral modiolar artery (Fig. 1A). The [Ca^2+^]_i _returned to almost resting levels after the transient increase, while the constriction was maintained. The ET-1-induced vasoconstriction was not readily reversible upon removal of ET-1 from the perfusate. The constriction and the increased vasomotion were observed without a significant change for at least 20 minutes after removal of ET-1 from the superfusate (*data not shown*). Note that ET-1-induced a transient [Ca^2+^]_i _increase and a sustained vasoconstriction while exposure to 10 mmol/l Ca^2+ ^induced an increase in [Ca^2+^]_i _and a parallel vasoconstriction.

We tested the potency of different Rho-kinase inhibitors reversing ET-1-induced constriction. Figure 1B shows an original recording of an ET-1-induced constriction which is antagonized by increasing concentrations of fasudil. Fasudil mediated vasodilation were induced without significantly altering [Ca^2+^]_i_-levels. The Rho-kinase inhibitors Y-27632, fasudil and hydroxy-fasudil reversed ET-1-induced constriction (10 nmol/L) in a dose-dependent manner (Figure 1C). The *1C_50 _*for Y-27632-, fasudil- and hydroxy-fasudil- mediated reversion of constriction was 3 μmol/L (p*IC_50 _*= 5.50 ± 0.31; n = 6), 15 μmol/L (p*IC_50 _*= 4.71 ± 0.13; n = 7) and 111 μmol/L (p*IC_50 _*= 3.95 ± 0.24; n = 6), respectively. The Ca^2+ ^sensitivity of the contractile apparatus was assessed as linear slopes obtained from correlations of [Ca^2+^]_i _and vascular diameter. ET-1 (100 pmol/L) increased the Ca^2+ ^sensitivity (-36 ± 9 versus -62 ± 13 μm/Ca^2+^, n = 8; Figure 1D), fasudil (3 μmol/L) prevented the ET-1-induced increase in the Ca^2+ ^sensitivity (-17 ± 3 versus -16 ± 2 μm/Ca^2+^, n = 8, Figure 1E). Taken together, these observations demonstrate that ET-1-induced constriction in the SMA is maintained by a Rho-kinase-mediated increase of the Ca^2+ ^sensitivity of the contractile apparatus, which can be effectively reversed by Rho-kinase inhibition.

### Exogenous, cell-permeable cAMP (dbcAMP) reverses ET-1-induced constriction and Ca^2+^sensitization

**Figure 2:**
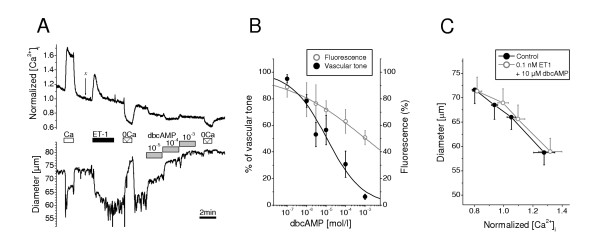
**dbcAMP reverses ET-1 (1 nmol/L) -induced vasoconstriction in a dose-dependent manner. A) **A representative recording of [Ca^2+^]_i_-diameter measurements. The [Ca^2+^]_i _was normalized to the value prior to application of ET-1 (value at timepoint "*x*" was set to 1.0). Maximal vasodilation was determined by exposure of the SMA to Ca-free conditions ("0Ca"). An increase of extracellular Ca^2+ ^to 10 mmol/L ("Ca") induced a [Ca^2+^]_i _increase and parallel vasoconstriction. **B) **Dose-response curves of dbcAMP-induced vasodilation and [Ca^2+^]_i _decrease of ET-1 (1 nmol/L) preconstricted SMA. C) dbcAMP prevented ET-1-induced Ca^2+^-sensitization.

DbcAMP induced dose-dependant decreases in [Ca^2+^]_i _and reversal of constriction induced by 1 nmol/L ET-1 with an *EC_50 _*of 1 mmol/L and 0.3 μmol/L (p*EC_50 _*= 2.97 ± 0.09 and 6.49 ± 0.07, n = 8, Fig. 2A and 2B), respectively. Note that dbcAMP-induced decreases in [Ca^2+^]_i _were less pronounced than those induced by removal of Ca^2+ ^from the extracellular solution although dilations were comparable (Fig. 2A). This observation suggests that the dilatory effect of cAMP is at least in part due to a decrease of the Ca^2+ ^sensitivity of the contractile apparatus. Consistent with this interpretation is the apparent rightward shift of the dose-response curve of dbcAMP for change in [Ca^2+^]_i _(Fig. 2B). If cAMP and ET-1 have opposing effects on the Ca^2+ ^sensitivity, it should be possible to prevent the ET-1-induced increase in the Ca^2+ ^sensitivity with dbcAMP. Indeed, 10 μmol/L dbcAMP prevented the increase in the Ca^2+ ^sensitivity induced by 100 pmol/L ET-1 (-29 ± 4 versus -27 ± 3 μm/Ca^2+^, n = 7; Figure 2C). These observations support the hypothesis that cAMP and Rho-kinase can interact as functional antagonists at the level of the Ca^2+ ^sensitivity of the contractile apparatus.

## Discussion

The primary observation of the present study is that the ET-1-induced SMA vasoconstriction is reversed by the Rho-kinase inhibitors Y-27632, fasudil and hydroxy-fasudil and by the cAMP analogue dbcAMP. All four agents decreased VSMC contractile apparatus Ca^2+ ^sensitivity.

In general, ET-1-induced constriction has been found to be elicited by different Ca^2+ ^mobilizing mechanisms, including Ca^2+ ^release from intracellular Ca^2+ ^stores via a phospholipase C mediated activation of IP_3_-receptors and activation of L-type and non-selective Ca^2+ ^channels [20-22]. Ca^2+ ^mobilization has been considered to be the main mechanism of ET-1-induced constriction. In contrast, according to our previous results, ET-1-induced Ca^2+ ^mobilization in the SMA appears to play a minor role [17]. The major mechanism of ET-1-induced constriction is an increase in the Ca^2+ ^sensitivity of the contractile apparatus [9].

The increase in the Ca^2+ ^sensitivity appears to be mediated by a Rho-kinase dependent inactivation of MLCP. This hypothesis is supported by two observations. First, inhibition of Rho-kinase with the selective Rho-kinase inhibitor Y-27632 abolished the ET-1-induced increase in the Ca^2+ ^sensitivity. Y-27632 at concentrations of up to 10 μmol/L has been shown to be a selective Rho-kinase inhibitor [23-25]. Second, inhibition of Rho-kinase with the selective Rho-kinase inhibitors fasudil and its functional metabolite hydroxy-fasudil also reversed ET-1-induced constriction and Ca^2+ ^sensitization of the contractile apparatus. These inhibitors have also been shown to be selective Rho-kinase inhibitors up to 20 μmol/L [24,26,27]. Since Y-27632 and the fasudil derivatives are structurally unique, but were observed to have similar functional effects, the concern of non-specific effects of the inhibitors can be minimized.

It has been shown that the Rho-kinase-dependent inhibition of the MLCP results from phosphorylation of the myosin-binding subunit (MBS) of the enzyme [28]. All Rho-kinase inhibitors employed in the present study reversed ET-1-induced constriction, with a clinically relevant *EC_50 _*range below the level reported to cause systemic side effects (especially hypotension). This possibly indicates that basal Rho-kinase activity is rather low under normal conditions [23,29]. Thus, an up-regulation of Rho-kinase expression/activity under pathophysiological conditions (hypertension, cerebral and coronary vasospasm) would impart Rho-kinase inhibitors with pharmacological relevance [29].

We show that cAMP is a potent vasodilating second messenger in the SMA, acting by mechanisms which decrease [Ca^2+^]_i _and Ca^2+ ^sensitivity. Although cAMP reduces intracellular Ca^2+ ^levels, the present data demonstrate that the main pathway targets the Ca^2+ ^sensitivity of the contractile apparatus. This interpretation is consistent with findings obtained in other preparations such as permeabilized intestinal and bronchial smooth muscle. In these studies the authors also could show that cAMP mediates a Ca^2+ ^desensitization of the contractile apparatus [30,31]. cAMP seems to be the main second messenger of CGRP-induced Ca^2+ ^desensitization and vasodilation in the SMA [18]. The data supports our hypothesis that the reversal of ET-1-induced constriction is not limited to inhibition of ET-1-related mechanisms (e.g., Rho-kinase signalling) and that ET-1-independent mechanisms can be targeted pharmacologically to reverse ET-1-mediated constriction. Clinical relevance arises from the possibility of modulating pharmacologically VSMC cAMP concentrations.

## Conclusion

Although the sudden loss of hearing causes substantial distress and pronounced long-term effects in affected individuals, adequate strategies to clinically treat the disorder are lacking. One subgroup of SSHL is believed to arise from SMA vasospasm(s) which ultimately lead to ischemic stroke of the inner ear [32]. The targeting of two distinct signalling mechanisms, Rho-kinase and cAMP, were both effective in reversing SMA constriction. Of note, two of the agents employed in the present study (fasudil and hydroxy-fasudil) are currently used in the clinical setting and were found to be effective at concentrations below the threshold of systemic side effects [8,33]. Thus, this study presents two novel, clinically relevant approaches for the treatment of SSHL. We therefore propose that clinical investigation into the use of these agents for SSHL treatment is warranted.

## Competing interests

The author(s) declare that they have no competing interests.

## Authors' contributions

EQS conducted all of the experiments and was involved in all aspects of data analysis and manuscript preparation. PW assisted with all facets of this study, assisting with data analysis and manuscript preparation. PW also provided laboratory space, equipment and financial support. WA provided financial support and made substantial contributions to conception and design of the present study.

All authors have been involved in drafting the manuscript and revising it critically for important intellectual content and given final approval of the version to be published.

## Pre-publication history

The pre-publication history for this paper can be accessed here:
